# Mutations causing low level antibiotic resistance ensure bacterial survival in antibiotic-treated hosts

**DOI:** 10.1038/s41598-018-30972-y

**Published:** 2018-08-21

**Authors:** Jakob Frimodt-Møller, Elio Rossi, Janus Anders Juul Haagensen, Marilena Falcone, Søren Molin, Helle Krogh Johansen

**Affiliations:** 1 Novo Nordisk Foundation Center for Biosustainability, Technical University of Denmark, Lyngby, Denmark; 2 Department of Clinical Microbiology, Rigshospitalet, Copenhagen, Denmark; 3 Department of Clinical Medicine, Faculty of Health and Medical Sciences, University of Copenhagen, Copenhagen, Denmark; 4 Present Address: Center for Peptide-Based Antibiotics, University of Copenhagen, Copenhagen, Denmark

## Abstract

In 474 genome sequenced *Pseudomonas aeruginosa* isolates from 34 cystic fibrosis (CF) patients, 40% of these harbor mutations in the *mexZ* gene encoding a negative regulator of the MexXY-OprM efflux pump associated with aminoglycoside and fluoroquinolone resistance. Surprisingly, resistance to aminoglycosides and fluoroquinolones of *mexZ* mutants was far below the breakpoint of clinical resistance. However, the fitness increase of the mutant bacteria in presence of the relevant antibiotics, as demonstrated in competition experiments between mutant and ancestor bacteria, showed that 1) very small phenotypic changes cause significant fitness increase with severe adaptive consequences, and 2) standardized phenotypic tests fail to detect such low-level variations. The frequent appearance of *P. aeruginosa mexZ* mutants in CF patients is directly connected to the intense use of the target antibiotics, and low-level antibiotic resistance, if left unnoticed, can result in accumulation of additional genetic changes leading to high-level resistance.

## Introduction

*Pseudomonas aeruginosa* is a frequent colonizer of the airways of cystic fibrosis (CF) patients, where it adapts easily and readily establishes chronic infections^1,2^. Treatment of *P. aeruginosa* lung infections is usually based on susceptibility testing of clinical isolates cultured from the patients’ airways secretions. In the initial period of colonization the aim is complete eradication of the infecting bacteria (*P. aeruginosa*) whereas in later stages the aim is at improving the lung function and diminish lung tissue damage, inflammation and thereby loss of lung function. However, the clinical outcome often does not confirm the importance of the intensive and frequent antibiotic treatment strategy, since in many cases the targeted bacteria persist in the patient airways despite their susceptibility to the administered drug^3^. For example, no correlation between antibiotic susceptibility to tobramycin and ceftazidime and clinical outcome has been reported^4^. A large number of *P. aeruginosa* isolates (n = 474) obtained from 34 young CF patients were genotyped by whole genome sequencing, and it was found that a small number of genes (n = 52) were targeted by patho-adaptive mutations, suggested to increase *P. aeruginosa* fitness during adaptation in the CF lungs^5^. Among these mutations, it was found that mutations in the *mexZ* gene were more frequent than any other patho-adaptive mutation in agreement with findings from other studies^6–11^.

MexZ is a negative regulator of the *mexXY* genes^12^, which together with *oprM* encode the MexXY-OprM efflux pump^13^. MexXY-OprM is one of four multidrug efflux systems of the resistance-nodulation-cell division family in *P. aeruginosa*^14^. The MexXY-OprM pump is often associated with active efflux of aminoglycosides and fluoroquinolones^15^, and recently aminoglycoside resistance in *P. aeruginosa* isolated from CF patients was reported to arise from convergent evolution in the *mexZ* gene^16^.

In many CF centers inhalation of tobramycin with or without *per oral* ciprofloxacin is one of the most important antibiotic therapies against initial and subsequent *P. aeruginosa* airway infections^17^. Moreover, tobramycin is one of the most frequently used drugs for intravenous (i.v.) treatment of chronically infected CF patients, and oral ciprofloxacin is used for maintenance therapy between i.v. courses to diminish inflammation and loss of lung function^18^. Because of the frequent use of aminoglycosides and fluoroquinolones in CF therapy there is a major selection pressure for increased tolerance to these antibiotics, arguably including constitutive overproduction of *mexXY* in *P. aeruginosa* during adaptation to the CF airways. Mutations in the genes PA5471.1, *armZ*, *mexZ*, and in the genes encoding the two-component system ParRS, have all been described to result in overproduction of *mexXY*^15^.

This study was initiated on the basis of (1) genomic investigations of 474 *P. aeruginosa* isolates from CF patients^5^, which showed that mutations in *mexZ* were more frequent that changes in any other gene; and (2) lack of correlation between these mutations and clinically relevant antibiotic resistance. It was therefore considered important to determine the phenotype of *mexZ* mutant strains in order to identify the selection pressure in CF airways, which could enrich for the occurrence and maintenance of *mexZ* bacterial cell lines.

## Results

### Mutations in *mexZ* identified in *P. aeruginosa* from airways of CF patients

**Figure 1:**
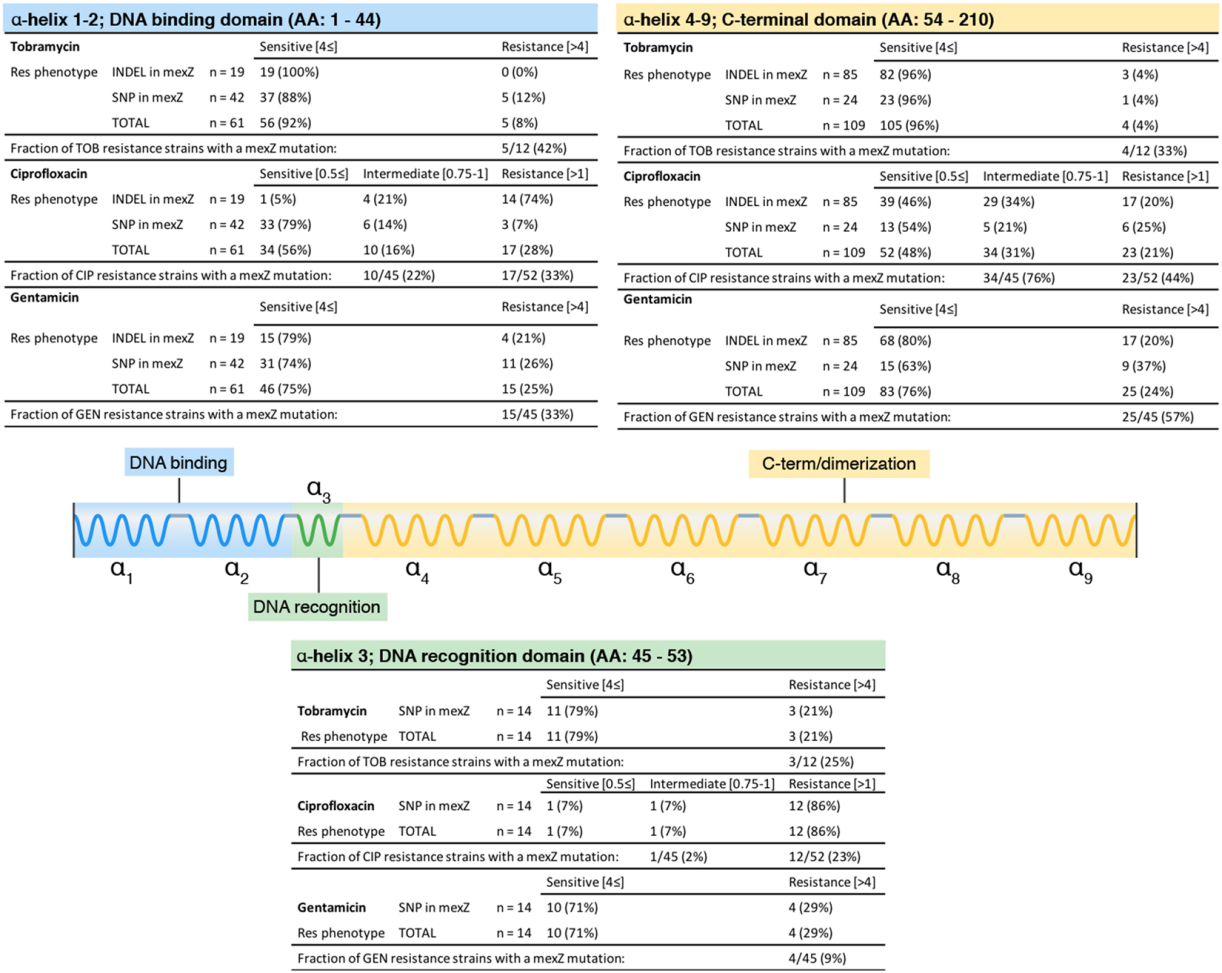
Mapping of clinical obtained mutations within the *mexZ* gene in *P. aeruginosa*. Here, *mexZ* mutations were mapped to one of the three functional *mexZ* domains; the DNA binding domain (α-helix 1 to α-helix 3), where α-helix 3 is the DNA recognition helix, and a C-terminal domain (α-helix 4 to α-helix 9)^19^. For each domain, the number of *mexZ* mutations consisting of a SNP or INDEL is noted with the accompanying MIC determination for the known MexXY-OprM substrates tobramycin, ciprofloxacin, and gentamicin. The clinical break-off points are presented according to EUCAST guidelines^21^.

Among the 474 genome sequenced *P. aeruginosa* isolates^5^ we identified 193 isolates harboring mutations in the *mexZ* gene. These isolates represent 20 of the 36 clone types isolated from 23 of the 34 CF patients from whom the isolate collection was obtained^5^. We identified 110 insertion or deletions (INDELs) and 83 single-nucleotide non-synonymous mutations (SNPs) constituting 40 unique sequence variants that were scattered throughout the gene coding sequence with no apparent mutational hot-spots (Supplementary Fig. S1 and Table S1). However, we did observe a slightly higher number of mutations localized in the C-terminal domain of MexZ in agreement with previous findings^16^. Interestingly, 30 of the 40 unique sequence variants were INDELs (Supplementary Fig. S1 and Table S1) similar to some of the previously published data^7^, and in contrast to other studies that have reported higher frequencies of SNPs^10^ (Supplementary Fig. S1). The clinical *mexZ* mutations in this study either resulted in a frameshift or a non-synonymous mutation (Supplementary Fig. S1 and Table S1). All the non-synonymous mutations mapped to each of the three domains of MexZ, in the proximity of genomic loci that have been previously connected with loss of MexZ function^19^. Thus, we expected that the vast majority of the reported clinical mutations in the *mexZ* gene lead to a complete loss-of-function and an increased expression of *mexXY*. Moreover, no direct association was found between type of mutation (SNP/INDEL), specific mutation locus, and the antibiotic resistance phenotype (Fig. 1). None of the 474 sequenced isolates harbored mutations in *parR* or PA5471.1, while one isolate had a mutation in PA5471, and eight isolates had mutations in *parS*. Six of the eight isolates containing a *parS* mutation also had a *mexZ* mutation. However, we found no correlation between multiple regulator mutations and resistance to any of the tested antibiotics. In addition, we found no evidence for *mexZ* being fixed together with any other specific mutations. These observations suggests that among the clinical *P. aeruginosa* isolates from CF patients at the Copenhagen CF Clinic, *mexZ* is by far the dominant target gene for mutations potentially affecting expression of *mexXY*. The strong selection for mutations in *mexZ* was further documented by fixation of the mutated alleles over time in the airway populations (Supplementary Figs S2 and S3).

### The regulatory phenotype of *mexZ* mutations

**Figure 2:**
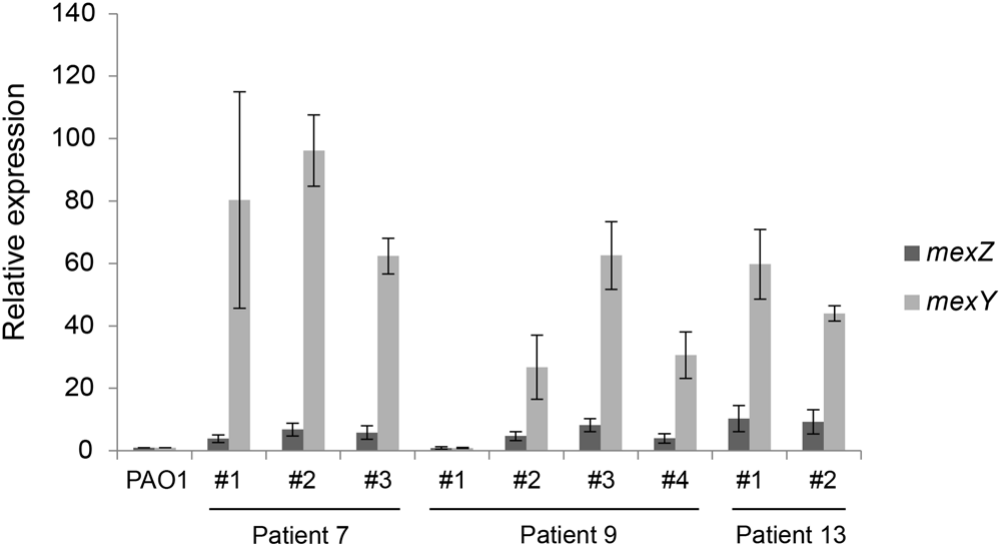
M*exZ* and *mexY* expression for selected clinical isolates. Relative expression of *mexZ* and *mexY* determined by RT-qPCR on RNA extracted from selected clinical isolates all of which contained a *mexZ* mutation apart from sample #1 from patient 9 (see Table S3 for details). All data were normalized to 16S RNA and ΔCt values between the genes of interest and 16S RNA were set at 1 for PAO1 wild-type strain. Experiments were performed in triplicate.

To investigate the phenotype of *mexZ* mutations in the clinical *P. aeruginosa* isolates, levels of MexY mRNA were determined from a number of representative strains isolated from 3 different CF patients (Fig. 2). In 8 out of 8 *mexZ* mutants of 3 different clone types harboring either missense mutations or deletions, an induction of the MexY mRNA was observed (more than 20 fold), whereas a strain with no mutation in *mexZ* expressed MexY mRNA at the low level similar to the wild-type. This suggests that all the different clinical *mexZ* mutations (both INDELs and SNPs) resulted in loss of function of the MexZ regulator. Interestingly, *mexY* expression varied greatly between the clinical *mexZ* mutants (from roughly 30 to 100 fold induction); we did not, however, find any correlation between high *mexY* expression and increased resistance to MexXY-OprM substrates (Fig. 2 and Supplementary Table S2).

Characterization of the functional consequences of mutations in the *mexZ* gene in the clinical strains is complicated due to the many other mutations accumulated in the genomes of these strains. Therefore, a PAO1 reference strain with a complete deletion of the *mexZ* gene was constructed for thorough phenotypic characterization of *mexZ* null-mutations without interference from other mutations in the genome (see details in Supplementary Information). We decided to delete the entire *mexZ* gene in PAO1 in order to construct a true knock-out variant and because we predict that the majority of the clinical INDELs and SNPs in our collection are likely to result in a functionally inactive MexZ protein (Fig. 2 and Supplementary Table S2), as observed in other works^9–11,16,19^.

**Figure 3:**
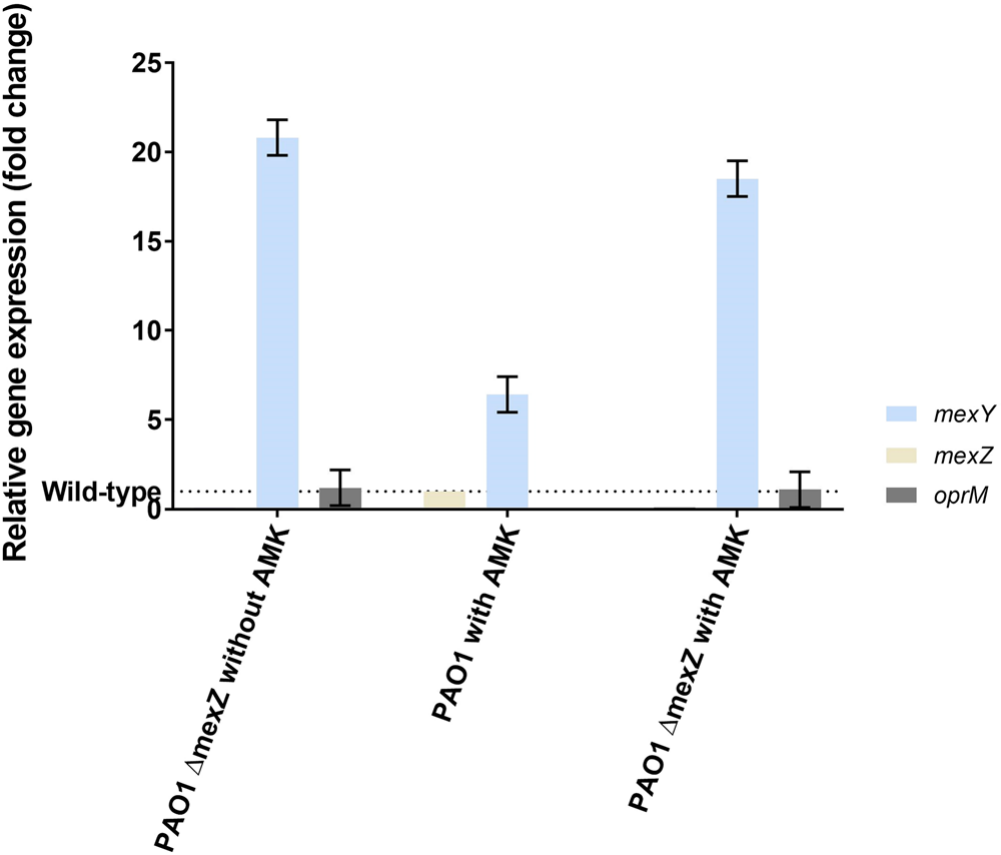
m*exY* and *oprM* expression. Comparative expression of the MexZ, MexY and OprM mRNA by RT–qPCR of PAO1 and PAO1 Δ*mexZ*, bacteria grown to exponential phase (OD_600_ = 0.5) in LB medium. Every experiment were related to PAO1 (wild-type) as indicated by the dash line. If amikacin was supplemented it was added at OD_600_ 0.1 at sub-MIC concentration (1 µg/mL). All data were normalized to the endogenous reference gene *rpsL*^15^. No *mexZ* expression was detected in experiments with PAO1 Δ*mexZ* and no bar is shown. Experiments were performed in triplicate.

Expression of the efflux pump operon *mexXY* in PAO1 wild-type and *mexZ* mutant bacteria was analyzed using qPCR (Fig. 3). Aminoglycosides are known to induce the expression of the *mexXY* operon^20^. Although the aminoglycoside tobramycin is used more frequently in the clinic to treat *P. aeruginosa* infections in CF patients we chose to use amikacin as a model aminoglycoside antibiotic for two reasons: (1) amikacin gave a stronger phenotype in MIC experiments (see next paragraph), and (2) amikacin has previously been used as a model aminoglycoside for investigations of the MexXY-OprM pump^16^, therefore making easier to compare our results with phenotypes deriving from previously documented mutations. We grew bacteria in LB broth in absence or presence of sub-MIC concentrations of amikacin and determined gene expression of *mexZ* and *mexXY-oprM* operon. When PAO1 Δ*mexZ* was grown without antibiotic, a roughly 20-fold increase in *mexY* transcription compared to the wild-type cells grown under the same conditions was observed (Fig. 3). As expected no *mexZ* transcripts were detected in PAO1 Δ*mexZ* under both growth conditions. PAO1 grown in the presence of sub-MIC concentrations of amikacin showed a 7-fold increase in *mexY* transcription compared to growth in the absence of amikacin (Fig. 3). Thus, as expected, the intrinsic response to aminoglycosides leads to an increased expression of *mexXY*^15^. PAO1 Δ*mexZ* grown in the presence of sub-MIC concentrations of amikacin only showed a 2.5-fold increase in *mexY* transcription compared to PAO1 grown under the same conditions (Fig. 3). This suggests that deletion of *mexZ* results in a maximal induction of *mexXY* (with or without antibiotics), and an overall 2.5-fold extra expression in the presence of amikacin. Interestingly, the OprM transcription level was unaltered in PAO1 Δ*mexZ* grown with or without the addition of amikacin (Fig. 3). These qPCR data confirm that inactivation of *mexZ* gene results in *mexXY* induction during growth.

To fully understand the consequences of mutations in the *mexZ* regulatory gene, we investigated global gene expression profiles of PAO1 wild type and *mexZ* mutant by genome-wide RNA sequencing. Cells were grown in LB without antibiotics, and RNA was extracted at OD_600_ 0.5. RNA sequencing showed little or no difference between the cellular transcriptomes of PAO1 and PAO1 Δ*mexZ*. Indeed, only three differentially expressed genes were identified as a result of the *mexZ* mutation: *mexX* and *mexY* transcription was induced, while *mexZ* mRNA levels were reduced, as expected (Table 1). These results confirm what was observed in qPCR analysis and clearly show that the MexZ regulator only affects expression of *mexXY* operon, without influencing any other transcriptional activities.

**Table 1:** Transcriptomic profiling of *mexZ* mutant strains. Cultures of PAO1 and PAO1 Δ*mexZ*, were grown to exponential phase (OD_600_ = 0.5) in LB medium.

	Gene	Function	Log_2_ (fold change)
PAO1 Δ*mexZ*	*mexZ*	Transcriptional regulator	−2.11
	*mexX*	Periplasmic efflux lipoprotein	+2.07
	*mexY*	Efflux protein	+2.20

For each strain, total RNA was extracted from at least three independent biological replicates. Genes preferentially expressed in the wild-type ancestor compared to their respective Δ*mexZ* mutants are indicated by negative log2 fold changes and genes preferentially expressed in Δ*mexZ* mutants compared to their respective wild-type ancestor are indicated by positive log2 fold changes.

### Antibiotic resistance levels in *mexZ* mutant strains

MIC values for the 474 clinical isolates of *P. aeruginosa* including 184 out of 193 *mexZ* mutants were determined using E-tests (Liofilchem®, Roseto degli Abruzzi, Italy) for 8 clinically relevant antibiotics including tobramycin and ciprofloxacin, and susceptibility/resistance definitions are according to the European Committee on Antimicrobial Susceptibility Testing (EUCAST) guidelines^21^ (Table 2). This analysis showed that the *mexZ* mutation only confers phenotypic resistance to few of the tested antibiotics. For instance, only 12 of the 184 (7%) investigated *mexZ* mutants were resistant to the MexXY-OprM substrate tobramycin, and 52 showed clinical resistance to ciprofloxacin (Table 2).

**Table 2:** MIC determination for clinical *P. aeruginosa* isolates *mexZ* mutations. MIC determined with E-tests (Liofilchem®, Roseto degli Abruzzi, Italy) on Müller-Hinton agar; Amikacin (AMK), Tobramycin (TOB), Gentamicin (GEN), Ceftazidime (CEF), Meropenem (MER), Aztreonam (AZT), Ciprofloxacin (CIP), and Colistin (COL).

Tobramycin			Sensitive [4≤]		Resistance [>4]
Res phenotype	INDEL in *mexZ*	n = 104	**101** (97%)		**3** (3%)
	SNP in *mexZ*	n = 80	**71** (89%)		**9** (11%)
	TOTAL	n = 184	**172** (93%)		**12** (7%)
Fraction of TOB resistance strains with a *mexZ* mutation:					**12/30** (40%)
**Ciprofloxacin**			**Sensitive [0.5≤]**	**Intermediate [0.75–1]**	**Resistance [>1]**
Res phenotype	INDEL in *mexZ*	n = 104	**40** (38%)	**33** (32%)	**31** (30%)
	SNP in *mexZ*	n = 80	**47** (59%)	**12** (15%)	**21** (26%)
	TOTAL	n = 184	**87** (47%)	**45** (25%)	**52** (28%)
Fraction of CIP resistance strains with a *mexZ* mutation:				**45/75** (60%)	**52/107** (49%)
**Gentamicin**			**Sensitive [4≤]**		**Resistance [>4]**
Res phenotype	INDEL in *mexZ*	n = 104	**83** (80%)		**21** (20%)
	SNP in *mexZ*	n = 80	**56** (70%)		**24** (30%)
	TOTAL	n = 184	**139** (76%)		**45** (24%)
Fraction of GEN resistance strains with a *mexZ* mutation:					**45/87** (52%)
**Colistin**			**Sensitive [4≤]**		**Resistance [>4]**
Res phenotype	INDEL in *mexZ*	n = 104	**104** (100%)		**0** (0%)
	SNP in *mexZ*	n = 80	**80** (100%)		**0** (0%)
	TOTAL	n = 184	**184** (100%)		**0** (0%)
Fraction of COL resistance strains with a *mexZ* mutation:					**0/0** (0%)
**Piperacillin**			**Sensitive [16≤]**		**Resistance [>16]**
Res phenotype	INDEL in *mexZ*	n = 104	**90** (87%)		**14** (13%)
	SNP in *mexZ*	n = 80	**50** (62%)		**30** (38%)
	TOTAL	n = 184	**140** (76%)		**44** (24%)
Fraction of PIP resistance strains with a *mexZ* mutation:					**44/90** (49%)
**Aztreonam**			**Sensitive [1≤]**	**Intermediate [1.5–16]**	**Resistance [>16]**
Res phenotype	INDEL in *mexZ*	n = 104	**32** (30%)	**64** (62%)	**8** (8%)
	SNP in *mexZ*	n = 80	**18** (23%)	**44** (54%)	**18** (23%)
	TOTAL	n = 184	**50** (27%)	**108** (59%)	**26** (14%)
Fraction of AZT resistance strains with a *mexZ* mutation:				**108/363** (30%)	**26/46** (57%)
**Ceftazidime**			**Sensitive [8≤]**		**Resistance [>8]**
Res phenotype	INDEL in *mexZ*	n = 104	**95** (91%)		**9** (9%)
	SNP in *mexZ*	n = 80	**64** (80%)		**16** (20%)
	TOTAL	n = 184	**159** (86%)		**25** (14%)
Fraction of CEF resistance strains with a *mexZ* mutation:					**25/65** (38%)
**Meropenem**			**Sensitive [2≤]**	**Intermediate [2.5–8]**	**Resistance [>8]**
Res phenotype	INDEL in *mexZ*	n = 104	**84** (80%)	**11** (11%)	**9** (9%)
	SNP in *mexZ*	n = 80	**61** (76%)	**10** (13%)	**9** (11%)
	TOTAL	n = 184	**146** (79%)	**21** (12%)	**18** (9%)
Fraction of MER resistance strains with a *mexZ* mutation:				**21/44** (47%)	**18/51** (35%)

MIC determinations for each antibiotic are given for the total population of *mexZ* mutants as well as the fraction of *mexZ* mutants with either an insertions/deletions (INDEL) or a Single-Nucleotide Polymorphism (SNP). In addition, the fraction of resistant strains with a *mexZ* mutation is given for each tested antibiotic. MIC values were compared to the clinical EUCAST breakoff points^21^.

The constructed PAO1 Δ*mexZ* mutant was also analyzed for its resistance to antibiotics using E-tests and the broth dilution method (Table 3). The chromosomal deletion of *mexZ* in PAO1 only affected the MIC of the already known MexXY-OprM substrates fluoroquinolones and aminoglycosides. Deleting *mexZ* in PAO1 resulted in slightly increased MIC values for all tested aminoglycosides as well as for ciprofloxacin. These slightly increased resistance levels were caused by induction of the MexXY-OprM pump, since cultures of PAO1 Δ*mexY* showed a decreased MIC value for the same compounds compared to the wild-type (Table 3). Increasing the OprM concentration in PAO1 Δ*mexZ*, using pXZL34 with an IPTG inducible OprM construct^22^, did not increase resistance to ciprofloxacin or amikacin (data not shown), suggesting that although OprM is expressed from the distal MexAB-OprM operon and is also required for the function of the MexAB efflux pump, the OprM level is not limiting for efflux pump activity when MexXY is fully induced and is sufficient to support the function of both the MexAB-OprM and the MexXY-OprM efflux pump. The differences in MIC between the mutants and their wild-type ancestor, PAO1, were in all cases minor, and none of the increased MIC values for PAO1 Δ*mexZ* could be categorized as ‘resistance’ based on the EUCAST break-off points for amikacin, tobramycin, gentamicin or ciprofloxacin^21^. When referring to MIC values in the following experiments we are referring to MIC values obtained only using the broth dilution method. The MIC determinations of the PAO1 Δ*mexZ* mutant are in agreement with the MIC data obtained from *P. aeruginosa* isolated from CF patients; i.e., there is no consistent correlation between a mutation in the *mexZ* gene and the clinical resistance to MexXY-OprM substrates. This may indicate that the in-patient selection pressure for *mexZ* mutations in *P. aeruginosa* may be driven by other factors than antibiotics.

**Table 3:** Wild-type and Δ*mexZ* phenotype(s).

AB MIC (µg/mL) E-test^a^	PAO1	PAO1 Δ*mexZ*	PAO1 Δ*mexY*
AMK	3	6	1
TOB	0.38	0.5	0.125
GEN	1.5	2	0.25
CEF	1.5	1.5	1.5
MER	0.5	0.5	0.5
AZT	3	3	3
CIP	0.064	0.19	0.047
COL	2	2	2
**AB MIC (µg/mL) BD** ^**b**^			
AMK	1	2	0.5
TOB	1	1	0.5
CIP	0.125	0.25	*N/A*
COL	2	2	*N/A*
ES MIC^c^			
H_2_O_2_ (mM)	0.16	0.16	*N/A*
Bile salt (%)	0.15	0.15	*N/A*
NaCl (mM)	0.5	0.5	*N/A*
**Doubling time (min)** ^**d**^			
LB	55	54	55
ASM	54	55	*N/A*
**Biofilm** ^**e**^			
LB	1.04	1.68	*N/A*
1 X M9 w. 0.2% GLU	0.91	0.96	*N/A*

^a^MIC determined with E-tests on Müller-Hinton agar. Amikacin (AMK), Tobramycin (TOB), Gentamicin (GEN), Ceftazidime (CEF), Meropenem (MER), Aztreonam (AZT), Ciprofloxacin (CIP), Colistin (COL).

^b^MIC determined using broth dilutions^34^. Antibiotic abbreviations are the same as above.

^c^Environmental stress MIC determined using broth dilutions^34^.

^d^Doubling time calculated from growth in LB, artificial sputum medium (ASM) or 1 X M9 w. 0.2% glucose. Cells were grown at 37 °C for 24 hours shaking.

^e^Biofilm measured by crystal violet staining on cells grown in either LB or 1 X M9 w. 0.2% glucose for 24 h in 96-well microtiter plates at 37 °C (no shaking).

Several environmental stress factors have been reported to interfere with the bacterial persistence in the airways of CF patients: increased levels of reactive oxygen species (ROS)^23^, bile aspiration caused by gastro-esophageal reflux^24^, and an abnormally increased Cl^-^ concentration^25^. ROS have been reported to induce *mexXY* expression in a PA5471 dependent fashion^26^. We therefore investigated whether a *mexZ* mutant exhibits an advantage over the wild-type if challenged by any of these stress factors. However, sensitivities to ROS, bile salt, and NaCl were equal for wild-type and *mexZ* mutants in all tested isolates, and none of these factors therefore seem to explain the positive selection for the *mexZ* mutation *in vivo* (Table 3)^26^. Furthermore, no difference was observed between the wild-type and Δ*mexZ* when grown in presence of multiple stresses: 0.5 X MIC ROS, 0.5 X MIC amikacin, and 0.5 X MIC NaCl (data not shown). When the concentrations of ROS, amikacin, and NaCl were increased no bacteria survived, as expected. Thus, no apparent differences in the ability to survive CF airway stress were observed when comparing wild-type and *mexZ* mutant bacteria.

### Growth physiology and catabolic properties of *mexZ* mutants

The apparent selective advantage of *mexZ* mutations could be the result of increased growth rates, improved capacities to utilize one or more carbon sources, or to form biofilm in sputum. Doubling times were determined both in LB and in ASM, which mimics the nutrient composition in CF lungs^27^. No significant growth rate differences were observed when comparing *mexZ* mutant and wild-type isolates, and biofilm development analyzed under two different growth conditions, LB and 1 X M9 minimal media with 0.2% glucose, also showed no measurable difference (Table 3).

We used the Omnilog Biolog (Biolog, Hayward CA, USA) system to monitor the catabolism of 190 different carbon sources for PAO1 and PAO1 Δ*mexZ* (Supplementary Fig. S4A,B). PAO1 and PAO1 Δ*mexZ* were able to catabolize the same carbon sources and we found no significant differences in growth when using these (Supplementary Fig. S4A,B).

### Fitness increase of *mexZ* mutant cells in presence of antibiotics

In order to assess the importance of the only phenotypic change observed for the *mexZ* mutant relative to its wild type ancestor, i.e. the very small increases in aminoglycoside and fluoroquinolone resistance, the growth and fitness properties of the two PAO1 strains in presence of sub-lethal concentrations of antibiotics were determined.

**Figure 4:**
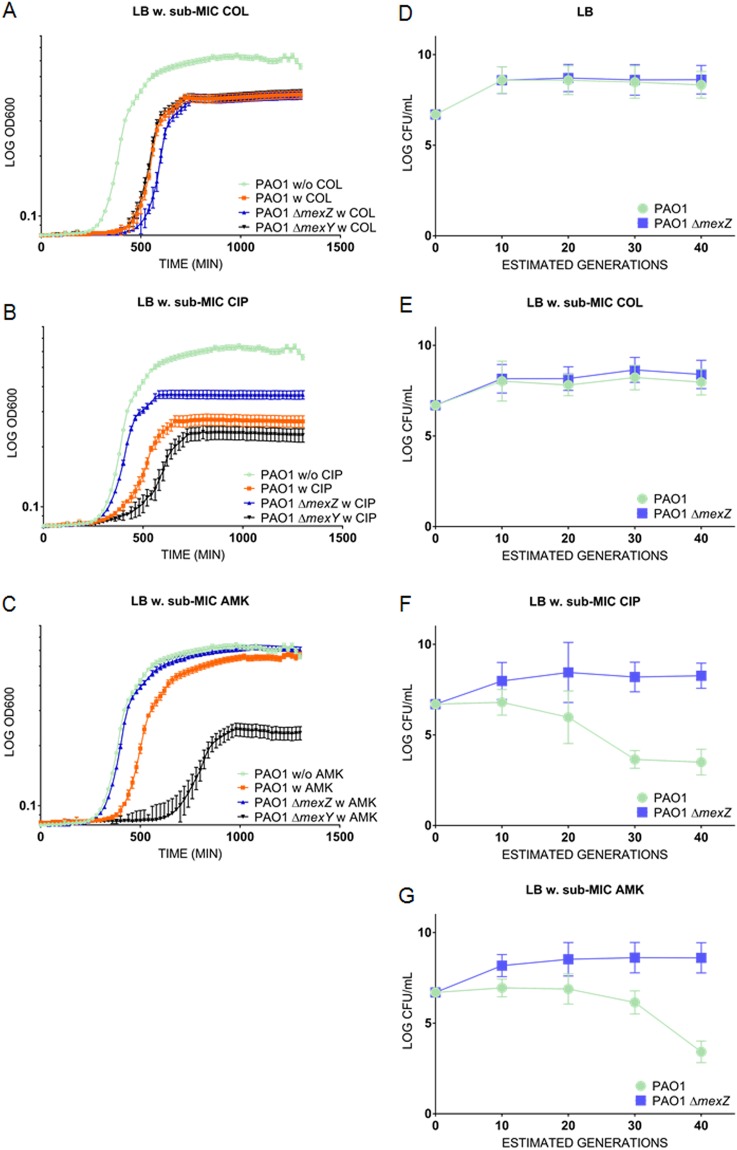
m*exZ* deficient bacteria has a faster recovery rate when challenged with *MexXY-OprM* substrates. In panel (A–C) we see antibiotic tolerance measured over time for PAO1, PAO1 Δ*mexZ*, and PAO1 Δ*mexY* to; (**A**) sub-MIC concentrations of colistin (1 µg/mL), (**B**) sub-MIC concentrations of ciprofloxacin (0.064 µg/mL), and (**C**) sub-MIC concentrations of amikacin (0.5 µg/mL). In (**A**,**B**), and (**C**) PAO1 was grown in the absence of colistin, amikacin, or ciprofloxacin, serving as a control. Panel (D) to (G) shows direct competition experiments between PAO1 Tn7:*gfp* (PAO1) and PAO1 Δ*mexZ*; (**D**) competition in LB in the absence of antibiotic, (**E**) competition in LB supplemented with sub-MIC concentrations of colistin (1 µg/mL), (**F**) competition in LB supplemented with sub-MIC concentrations of ciprofloxacin (0.125 µg/mL), (**G**) competition in LB supplemented with sub-MIC concentrations of amikacin (1 µg/mL). Bars represent the standard error of the log10 mean number of CFU per ml.

First, we investigated whether *mexZ* deficient cells could have an advantage when growing in medium with relevant antibiotics at, or close to, the MIC breakpoint. In presence of sub-MIC concentrations of the non-MexXY-OprM substrate, colistin, all strains (PAO1, PAO1 Δ*mexZ*, and PAO1 Δ*mexY*) showed identical growth phenotypes (Fig. 4A). However, when grown in medium with amikacin or ciprofloxacin (Fig. 4B,C) *mexZ* deficient cells showed a growth phenotype similar to wild-type cells grown without antibiotics, suggesting that in these conditions the *mexZ* mutant has a fitness increase over the wild type strain. This specific growth phenotype depends on a functional efflux-pump, since PAO1 Δ*mexY* shows a reduced growth rate compared to that of the wild-type and PAO1 Δ*mexZ* in media with amikacin or ciprofloxacin (Fig. 4B,C).

**Figure 5:**
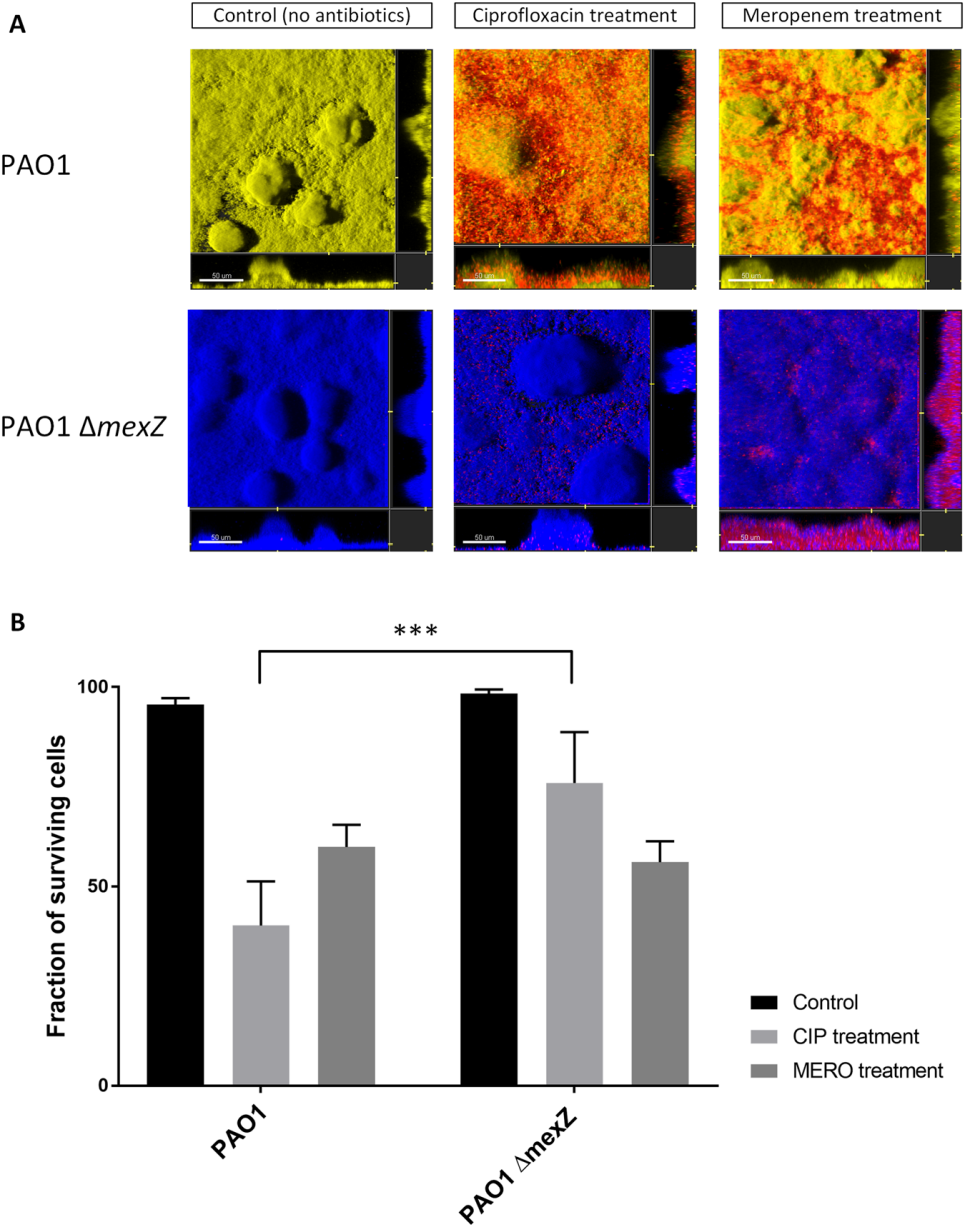
Survival of PAO1 wild-type and PAO1 Δ*mexZ* during treatment of biofilms with ciprofloxacin and meropenem. (**A**) Representative images of PAO1 tagged with yellow fluorescent protein and PAO1 Δ*mexZ* tagged with cyan fluorescent protein after live/dead staining (dead cells were stained for visualization using propidium iodide). Biofilms were either untreated, treated with one bolus (4 mg/l) Ciprofloxacin treatment for 24 h, or one bolus (107 mg/l) Meropenem for 24 h. Biofilms were grown on glucose minimal medium and treated with antibioitcs at day 3 (see Material and Methods). (**B**) COMSTAT quantification of the biomass of live and dead cells after live/dead staining of a PAO1 wild-type biofilm and PAO1 Δ*mexZ* biofilm from the same treatment regime as described in (**A**). In at least two independent experiments 3–4 random images were taken from each flow channel. A t-test (two-tailed) was performed between PAO1 and PAO1 Δ*mexZ* treated with ciprofloxacin showing a highly significant difference in survival (p < 0.001) as indicated by the three ***.

Second, we investigated PAO1 and PAO1 Δ*mexZ* in a flow-cell biofilm system developed for pharmaco-kinetic and -dynamic investigations with dosing of antibiotics according to the treatment regimes of CF patients in the clinic. Because ciprofloxacin is primarily used as maintainance therapy between i.v. courses in the clinic^18^, i.e. used as frequently as aminoglycosides if not even more (Supplementary Fig. S2), it was selected to illustrate a MexXY-OprM substrate (and meropenem as a non-substrate for same pump) in the flow-cell biofilm system. Each of the two strains (tagged with yellow fluorescent protein (YFP) and cyan fluorescent protein (CFP), respectively) were allowed to establish biofilms for 3–4 days in the flow cells before antibiotics were administered (see Material and Methods for details). After treatment with antibiotics for 24 hours the biofilms were stained with ‘dead’ stain propidium iodide (PI) and analyzed in the confocal microscope (Fig. 5A). The individual biomasses and dead-cell populations were determined by the quantitative COMSTAT analysis (Fig. 5B). Figure 5 clearly shows that the *mexZ* mutant bacteria signifincantly better (t-test (two-tailed); p-value < 0.001) tolerate the bolus of ciprofloxacin than the wild-type bacteria. This is particularly interesting because the *mexZ* mutant survives, even though concentrations of ciprofloxacin above MIC were administered to the biofilm cultures, and despite the very moderate difference in MIC between PAO1 and PAO1 Δ*mexZ*. In a parallel biofilm experiment, in which meropenem (not excreted by the MexXY-OprM efflux pump) was administered to the two bacterial populations, no difference in survival of the bacteria was observed (Fig. 5). These results show that *mexZ* mutant bacteria survive better in clinically relevant antibiotic containing environments, resulting (1) in their competitive advantage in populations comprising both wild type and MexZ mutant bacteria, and (2) fixation of the specific mutation.

Next, we tested the *mexZ* associated fitness increase in competition experiments including equal numbers of wild type and *mexZ* mutant bacteria (tagged differentially with fluorescent proteins) in liquid cultures in shaking flasks with low concentrations of antibiotics. In presence of amikacin or ciprofloxacin (sub-MIC concentrations) growing for more than 40 generations in direct competition, the *mexZ* mutant bacteria eventually out-competed the wild-type bacteria (Fig. 4F,G). When grown in LB or in LB supplemented with sub-MIC concentrations of colistin (which is not excreted by the MexXY-OprM efflux pump), both strains were stably maintained at the same ratio (Fig. 4D,E). These results show that *mexZ* mutations confer increased fitness in environments containing relevant antibiotics, and that in absence of these antibiotics mutant and wildtype cells are equally fit.

## Discussion

Treating patients suffering from persistent bacterial infections with antibiotics relies to a great extent on careful monitoring of resistance development. In line with this need, standardized protocols for clinical antibiotic susceptibility testing have been developed together with approved values for resistance break points (EUCAST). The obvious goal of course is to treat health threatening infections with antibiotics, which are assumed to be effective, and to avoid resistance development as much as possible. Previous studies have, however, shown a lack of correlation between clinical antibiotic susceptibility testing and clinical outcome^3,4^. The striking finding of this study is that very small increases in antibiotic resistance associated with *mexZ* mutations, which normally are undetected or neglected in the clinical microbiological laboratory using current standardized diagnostic methods, seem to be drivers of selection of *mexZ* mutants in CF patients. When *P. aeruginosa* colonizes the CF airways the bacteria are challenged with different stress factors, which may interfere with persistence, such as presence of antibiotics, increased levels of ROS^23^, bile aspiration caused by gastro-oesophageal reflux^24^, and an abnormally increased Cl^−^ concentration^25^. Surprisingly, however, we did not observe any marked difference in susceptibility to any of these stress factors, when comparing wild type and *mexZ* mutant under standard laboratory conditions. In addition, the *mexZ* mutants did not show any change in growth rate nor improved potential to form biofilm. However, when MexZ deficient bacteria were competed against wild type bacteria in presence of sub-MIC concentrations of amikacin or ciprofloxacin, the mutant bacteria quickly outcompeted the wild type. This was true both in direct competition in LB Broth (Fig. 4), and in our flow-chamber biofilm system, which mimics the *P. aeruginosa* biofilm lifestyle and the antibiotic treatment used in the CF clinic^28^, the mutant bacteria survived better than the wildtype bacteria (Fig. 5A,B). These results show that *mexZ* deficient bacteria are superior to the wild-type strains in clinically relevant sub-MIC growth conditions, which most likely are found in the lungs and sputum of CF patients during antibiotic treatment.

Since aminoglycosides and fluoroquinolones are very important first-line drugs for treating *P. aeruginosa* airway infections in CF patients, it is of major importance that changes in antibiotic susceptibility are identified in the clinical microbiological laboratory. However, the differences in aminoglycoside and fluoroquinolone MIC values for the *mexZ* mutant relative to those of the wild-type ancestor, PAO1, were in all cases minor, and none of the increased MIC values for PAO1 Δ*mexZ* could be categorized as resistance based on the EUCAST break-off points for amikacin, tobramycin, gentamicin or ciprofloxacin^21^. The discrepancy between the accumulation of *mexZ* mutations, and the lack of a clear antibiotic resistance phenotype when standard EUCAST methods for antibiotic resistance assessments are used^21^, creates a clinical problem since the fitness increases of the mutant bacteria in presence of the relevant antibiotics remain undetected, and therefore treatment may erroneously be continued. In fact, all 13 CF patients listed in Supplementary Fig. S2 received tobramycin and/or ciprofloxacin treatment after the fixation of *mexZ* mutations in the respective *P. aeruginosa* populations.

Genetic changes increasing even slightly the fitness of an infecting bacterial populations under conditions of antibiotic treatment may also increase the chance of acquisition of new mutations making the bacteria truly resistant to the respective antibiotics. Recently, an investigation of antibiotic resistance development in *Mycobacterium smegmatis* documented how mutations in genes encoding ribosomal components, resulting in low-level resistance to one or more antibiotics, may promote the subsequent acquisition of mutations resulting in high-level antibiotic resistance^29^. We suggest that *mexZ* mutations in a similar but mechanistically different way, may create evolutionary opportunities for the mutant subpopulations to evolve high-level resistance (cf. Supplementary Fig. S3; Patient 1). In addition, we also observed clonal interferences between competing lineages with different beneficial *mexZ* mutations (cf. Supplementary Fig. S2; Patient 5). Here, three different *mexZ* mutations approached fixation simultaneously, with one of them eventually being the sole *mexZ* mutation isolated from this patient for the entire study period.

Although our *mexZ* investigation is relevant for the chronic bacterial persistence in the upper airways in CF patients, the basic idea that beneficial mutations can evade clinical microbial diagnostics can be extrapolated to other genes in unrelated infection scenarios. It is imperative for optimal and effective antibiotic treatments of chronic bacterial infections that the diagnostic determination of antibiotic susceptibility is correct and constitutes a reliable information base for designing a treatment strategy for the patient. If the clinical microbiology laboratory only determines antibiotic susceptibility of bacteria according to the standardized guidelines, it is highly likely that relevant mutations conferring phenotypic low-level antibiotic resistance will be ignored, which may have important consequences for the efficacy of the treatment, as reported here for the *mexZ* mutations in *P. aeruginosa*. Although the treatment strategies have improved significantly during the last decades, resulting in prolongation of the life span of CF patients^17^, there is room for further development of even more effective antibiotic regimens delaying the chronic infection state of the most problematic bacterial infections such as those caused by *P. aeruginosa*^30^. For CF patients based on the discordance between the clinical antibiotics susceptibility test results and the clinical outcomes, we suggest that targeted genotypic investigations may positively complement routine susceptibility testing, informing about genetic changes, which may have predictive impacts on the non-detectable resistance profile and associated persistence of colonization of the airway population.

## Material and Methods

### Growth conditions

Clinical isolates of *P. aeruginosa* were grown in Lysogeny Broth (LB) medium, 1 X M9 minimal medium supplemented with 0.2% glucose, or in artificial sputum medium (ASM)^27^. All liquid cultures were incubated at 37 °C with shaking (200 r.p.m.) unless otherwise stated. *Escherichia coli* was cultured on LB agar, and *P. aeruginosa* was cultured on *Pseudomonas* Isolation Agar (PIA; Sigma-Aldrich Co. LLC., St. Louis, USA) or LB agar. When necessary, antibiotics were added to the following concentrations: gentamicin, 100 µg/ml for *P. aeruginosa* and 10 µg/ml for *E. coli*; chloramphenicol, 20 µg/ml; ampicillin, 150 µg/ml; kanamycin, 50 µg/ml.

### Bacterial strains and plasmids

All strain and plasmid constructs are listed in Supplementary Materials and methods and in Supplementary Table 1. Oligonucleotides for PCR are listed in Supplementary Table 2. In addition, we used a collection of 474 genome sequenced *P. aeruginosa* isolates from 34 CF children and young adults to measure the minimal inhibition concentration (MIC), using relevant antibiotics, for clinical strains with- and without a *mexZ* mutation.

### Total RNA isolation

For RNA-seq and quantitative real-time-PCR (qRT-PCR) experiments, cultures were grown to exponential phase (OD600 = 0.5) in LB medium. For each strain, total RNA was extracted from at least three independent biological replicates using Trizol reagent (Thermo Fisher Scientific Inc.) followed by RNA clean & concentrator kit (Zymo Research, Irvin, USA) accordingly to vendors’ protocols. RNA quality was checked using RNA Nano kit on an Agilent Bioanalyzer 2100 machine. Samples with an RNA integrity number (RIN) greater than 9 were used in downstream analysis.

### qRT-PCR

For qRT-PCR experiments, after genomic DNA digestion, 1 µg of total RNA was retrotranscribed using QuantiTect Reverse Transcription kit (Qiagen, Venlo, Netherlands). Primers designed to amplify *mexZ*, *mexY*, and *oprM* (Supplementary Table 2) were targeted to regions of unique sequences within the genes in wild-type PAO1. The qRT-PCR was performed using the QuantiTect SYBR® Green PCR Kit (Qiagen, Venlo, Netherlands) on an Agilent Stratagene Mx3000P qPCR system. All data were normalized to either the 16S RNA (Fig. 2) or the endogenous reference gene *rpsL*^15^ (Fig. 3). These data were transformed to log2 to obtain a change difference (n-fold) between strains.

### RNA sequencing and data analysis

For RNA-sequencing experiments, 10 µg of total RNA was depleted of ribosomal RNA using Ribo Zero rRNA removal kit for Gram-negative bacteria (Illumina). Strand-specific sequencing libraries were prepared using 50 ng of mRNA-enriched samples as input for TruSeq stranded mRNA library preparation kit (Illumina) following vendor’s recommendations. Sequencing was performed on an Illumina NextSeq 500 system to a depth of 15–20 million reads per samples. After quality filtering, raw reads were aligned using BWA aligner against the relevant genome (*P. aeruginosa* PAO1: NC_002516.2). Read counts for gene relative abundance were obtained using HTSeq-count tool from HTSeq package^31^,while gene differential expression and statistical analysis were performed as previously described^32^. The transcriptomic data have been submitted to the ArrayExpress database (http://www.ebi.ac.uk/arrayexpress/) and assigned the identifier E-MTAB-5820.

### Optical density measurements over time

The stress resistance assay was performed as previously described^33^. In short, the overnight cultures were diluted to a final desired inoculum of 5 × 10^5^ cfu/mL, as described by Wiegand *et al*.^34^, with and without the antibiotic, and growth was monitored at OD_600_ in 96-well microtitre polystyrene plates for 20–24 h. The concentration of the antibiotic was adjusted to avoid killing of the bacteria; i.e. no visible growth after 24 hours. Here we used amikacin (0.5 µg/mL), ciprofloxacin (0.064 µg/mL), and colistin (1 µg/mL).

### Minimal inhibition concentration measurements (MIC)

#### E-test

E-tests were performed with Mueller-Hinton agar plates (diameter, 140 mm) as previously described^35^. The direct colony suspension method was used to make a suspension of PAO1, PAO1 Δ*mexZ* and PAO1 in saline to the density of McFarland 0.5 turbidity standard, approximately corresponding to 1–2 × 10^8^ CFU/mL for *E. coli*. E-tests (Liofilchem®, Roseto degli Abruzzi, Italy) for clinical relevant antibiotics were used; amikacin, tobramycin, gentamicin, ciprofloxacin, colistin, ceftazime, meropenem, and aztreonam. After application of the E-test, the plates were incubated at 37 °C for 16–20 h. MIC values were compared to the clinical EUCAST breakoff points^21^.

#### Broth micro-dilution

Broth micro-dilutions were performed in 96-well microtiter plates (polystyrene plates (BD Falcon; Fisher Scientific, cat. no. 351177)) as previously described with the exception that dilutions were performed in LB broth, not in Mueller-Hinton broth. Broth micro-dilutions were done for the clinically relevant antibiotics (all from Sigma-Aldrich Co. LLC.) amikacin, tobramycin, ciprofloxacin, and colistin. In addition, the MICs with respect to hydrogen peroxide (Thermo Fisher Scientific Inc.), NaCl (Thermo Fisher Scientific Inc.), and bile salts (Oxgall; Neogen Corporation, Lansing, USA) were tested using the same protocol. After addition of antibiotic or stress factor, plates were incubated at 37 °C for 16–20 h. The MIC was defined as the lowest concentration of the antimicrobial agent or stress factor that inhibits visible growth of the tested isolate as observed with the unaided eye^34^.

### Biolog Phenotype Microarrays

Biolog Phenotype Microarrays were performed as previously described^36^. In short, PAO1 and PAO1 Δ*mexZ* strains were streaked on LB agar plates and incubated at 37 °C. Cells were swabbed from the plates and suspended in IF-0 GN Base (inoculation fluid) at a density corresponding to 42% transmittance in the Biolog turbidimeter. The cell suspensions were diluted in IF-0 minimal medium containing Biolog redox dye mixture D (tetrazolium), and aliquots were added to the two different carbon-source plates; PM1 and PM2A. The plates were incubated at 37 °C in an OmniLog plate reader (Biolog) for 48 h, and growth/respiration was measured kinetically by determining the colorimetric reduction of the tetrazolium dye. Export of OmniLog data was performed using OmniLog *OL_FM/Kin* 1.20.02 software (Biolog). The average area beneath each kinetic curve was used for analysis. Total catabolic function was calculated as previously described^37^.

### Static biofilms

Biofilm formation in micro-titre plates was analysed as previously described^38^. PAO1 and PAO1 Δ*mexZ* were grown in either LB or 1 X M9 with 0.2% glucose overnight at 37 °C and diluted into identical media for the study of biofilm formation. The biofilm was stained by 0.1% solution of crystal violet (Thermo Fisher Scientific Inc.) and quantified in a plate reader (OD550 nm) using the growth media without added bacteria as the blank.

### Pharmacodynamic/pharmacokinetic (PD/PK) flow chamber biofilms

The PD/PK system is based on bacterial biofilms grown under continuous culture conditions, simulating the changing antibiotic concentrations in CF patients during i.v. dosing. In this system the antibiotics decay similarly to what takes place in CF patients during treatment. Antibiotic treatment of biofilms were performed using differentially tagged strains, i.e. PAO1 tagged with yellow fluorescent protein (YFP) and PAO1 Δ*mexZ* tagged with cyan fluorescent protein (CFP). Dead cells were stained for visualization using propidium iodide (see Supplementary Material and methods for strain constructions). The one-compartment dynamic biofilm PD model has been previously described^28^. Briefly, biofilms were grown at 30 °C in flow chambers for 96 hours, at which point ciprofloxcacin or meropenem concentrations modeling human PK were applied. Each flow chamber was inoculated with 250 μL of overnight cultures of PAO1 or PAO1 Δ*mexZ* respectively diluted to an OD_600_ of 0.05 and left without flow for one hour. After one hour, flow was started with 40x diluted LB or minimal medium supplemented with 0.03 mM glucose at a flow rate of 20 ml/h using a peristaltic pump (Watson Marlow 205S). After cultivation for 72 hours, flow was stopped and medium was replaced with an antibiotic flask containing relevant concentrations of either ciprofloxacin or meropenem. Flow was restarted and medium was pumped from the dilution flask through the antibiotic flask to the flow chambers at a constant rate calculated to mimic the elimination rate constant of the antibiotic. Concentration-time profiles were based on PK parameters of ciprofloxacin and meropenem from healthy people and patients with CF^39,40^.

The target ciprofloxacin peak concentration, based on human population values, was calculated to be 400 mg IV = 4 mg/L, and for meropenem it was 107.5 mg/L. Using these target concentrations the model allows for the simulation of the human PL profile of the antibiotic while maintaining constant flow rate. Experiments were repeated 3 times in biological independent runs, and 3–4 images of each channel were collected from each run using a Zeiss LSM 510 meta confocal laser scanning microscope (CLSM) equipped with an argon/krypton laser and detectors and filter sets for simultaneous monitoring of CFP (excitation 458 nm, emission 490 nm) and YFP (excitaiotn 514 nm, emission 530 nm) for live cells, and propidium iodide, PI (excitation 543 nm, emission 565 nm) for dead cell staining. Images were obtained using a 40x/1.3 Plan-Neofluar oil objective. Multichannel simulated fluorescence projections (SFPs) and sections through the biofilms were generated using Imaris software (Bitplane AG, Switzerland). CLSM images were analyzed using COMSTAT^41^.

### Ethics approval

The local ethics committee at the Capital Region of Denmark (Region Hovedstaden) approved the use of the stored *P. aeruginosa* isolates: registration number H-4-2015-FSP. We confirm that all methods were performed in accordance with the relevant guidelines and regulations.

## Electronic supplementary material

Supplementary material and methods
