# Supplementary material for: Mutations causing low level antibiotic resistance ensure bacterial survival in antibiotic-treated hosts
Source: Sci Rep. 2018 Aug 21;8:12512. doi: 10.1038/s41598-018-30972-y (PMC6104031; doi:10.1038/s41598-018-30972-y)

**ORIGINAL ARTICLE**

**TITLE**

Mutations causing low level antibiotic resistance ensure bacterial survival in antibiotic-treated hosts

**AUTHORS**

Jakob Frimodt-Møller^1^, Elio Rossi^2^, Janus Anders Juul Haagensen^1^, Marilena Falcone^2^, Søren Molin^1*^, and Helle Krogh Johansen^2,3*^

**Supplementary material and methods**

**Bacterial strains and plasmids**

*Plasmids for chromosomal deletion of mexZ and mexY in PAO1*

The deletion construct was created by USER cloning and USER fusion as previously described ^1^ in pEX19Gm. All PCR amplifications were amplified using Phusion U Hot Start DNA Polymerase (Thermo Fisher Scientific Inc., Waltham, USA). Flanking sequences of the *mexZ*- and *mexY* gene were amplified by PCR from PA01 chromosomal DNA using primers corresponding to the PA2020 and PA2018 predicted ORF sequence, respectively. The first round of PCR created two products using the following primers. For Δ*mexZ* in PAO1: *mexZ*_PAO1_UP_Fw and *mexZ*_PAO1_UP_Rv; *mexZ*_PAO1_DW_Fw and *mexZ*_PAO1_DW_Rv. For Δ*mexY* in PAO1: *mexY*_PAO1_UP_Fw and *mexY*_PAO1_UP_Rv; *mexY*_PAO1_DW_Fw and *mexY*_PAO1_DW_Rv. The second round of PCR used the following primers with pEX19Gm as the template. For Δ*mexZ* in PAO1: pEX19Gm_*mexZ*_PAO1_Fw and pEX19Gm_*mexZ*_PAO1_Rv; for Δ*mexY* in PAO1: pEX19Gm_*mexY*_PAO1_Fw and pEX19Gm_*mexY*_PAO1_Rv. The PCR products were purified using a PureLink™ Quick Gel Extraction and PCR Purification Combo Kit (Thermo Fisher Scientific). 100 ng of each fragment (flanking sequences and pEX19Gm PCR amplification) for *mexZ* and *mexY* in PAO1, respectively, was mixed in a total volume of 10 μL and buffered using the 5 X Phusion HF Buffer (Thermo Fisher Scientific). For template removal, *DpnI* (Thermo Fisher Scientific Inc.) was added prior to USER™ enzyme mix and incubated for 1 h at 37°C, deactivated by incubation at 80°C for 5 min, and rested on ice for 5 min. 1 μL of USER™ enzyme mix (New England Biolabs, Ipswich, USA) was subsequently added to the reaction tubes, and uracil excision is accomplished by incubating the sample at 37°C for 15 min. DNA assembly is executed by cooling down the reaction to below the melting temperature of the cohesive ends for at least 15 min. For Δ*mexZ* in PAO1, pEX19Gm contains 1,027 bp from the 5’ region of the gene and 1,088 bp from the 3’ region the gene inserted into the multiple cloning site (MCS) creating pJFM20 (pEX19Gm:*mexZ*:PAO1). For Δ*mexY* in PAO1, pEX19Gm contains 693 bp from the 5’ region of the gene and 561 bp from the 3’ region the gene inserted into the MCS creating pJFM21 (pEX19Gm:*mexY*:PAO1). Chemical competent *E. coli* DH5ɑ were transformed with the USER fusion mixture and incubated for 1 h at 37°C and plated on LB plates supplemented with gentamycin and left O/N at 37°C. All plasmids were verified by DNA sequencing.

*Chromosomal deletion of mexZ and mexY in PAO1*

A broad-host-range Flp-*FRT* recombination system for site-specific excision of chromosomally-located DNA sequences to give unmarked *P.* *aeruginosa* mutants was used ^2^. Here, by the help of pRK600 (carrying the RP4/RP2 conjugation system), pJFM20 and pJFM21 was mated into PAO1^2^ to create PAO1 Δ*mexZ* and PAO1 Δ*mexY*, respectively. All deletions were verified by DNA sequencing.

*Site-specific tagging of with GFP, YFP, or CFP*

Here, we used the mini-Tn7 transposon system to site-specific tag PAO1 with GFP in the Tn7-site as previously described by Lambertsen *et al.* ^3^. In short, four-parental mating was used to mate either pBK-mini-Tn7-*gfp2* or pBK-mini-Tn7-*yfp* into PAO1 and pBK-mini-Tn7-*cfp* into PAO1 Δ*mexZ* with the help of pRK600 and pUX-BF13, which has an R6K origin and contains the Tn7 transposase genes (*tnsABCDE*) for insertion of mini-Tn7 in the chromosomal Tn7-site, creating PAO1 Tn7:*gfp,* PAO1 Tn7:y*fp*, and PAO1 Δ*mexZ* Tn7:*cfp*, respectively. GFP, YFP, and CFP are expressed from the constitutive *E. coli* *lac*-promoter derivative P_A1/04/03_ which has no influence on cellular growth ^3^.

**References**

1 Cavaleiro, A. M., Nielsen, M. T., Kim, S. H., Seppala, S. & Nørholm, M. H. H. Uracil Excision for Assembly of Complex Pathways. *Springer Protocols Handbooks*, doi:DOI 10.1007/8623_2015_133 (2015).

2 Hoang, T. T., Karkhoff-Schweizer, R. R., Kutchma, A. J. & Schweizer, H. P. A broad-host-range Flp-FRT recombination system for site-specific excision of chromosomally-located DNA sequences: application for isolation of unmarked Pseudomonas aeruginosa mutants. *Gene* **212**, 77-86 (1998).

3 Lambertsen, L., Sternberg, C. & Molin, S. Mini-Tn7 transposons for site-specific tagging of bacteria with fluorescent proteins. *Environ Microbiol* **6**, 726-732, doi:10.1111/j.1462-2920.2004.00605.x (2004).

4 Grant, S. G., Jessee, J., Bloom, F. R. & Hanahan, D. Differential plasmid rescue from transgenic mouse DNAs into Escherichia coli methylation-restriction mutants. *Proc Natl Acad Sci U S A* **87**, 4645-4649 (1990).

5 Holloway, B. W. & Morgan, A. F. Genome organization in Pseudomonas. *Annu Rev Microbiol* **40**, 79-105, doi:10.1146/annurev.mi.40.100186.000455 (1986).

6 Kessler, B., de Lorenzo, V. & Timmis, K. N. A general system to integrate lacZ fusions into the chromosomes of gram-negative eubacteria: regulation of the Pm promoter of the TOL plasmid studied with all controlling elements in monocopy. *Mol Gen Genet* **233**, 293-301 (1992).

7 Bao, Y., Lies, D. P., Fu, H. & Roberts, G. P. An improved Tn7-based system for the single-copy insertion of cloned genes into chromosomes of gram-negative bacteria. *Gene* **109**, 167-168 (1991).

8 Koch, B., Jensen, L. E. & Nybroe, O. A panel of Tn7-based vectors for insertion of the gfp marker gene or for delivery of cloned DNA into Gram-negative bacteria at a neutral chromosomal site. *J Microbiol Methods* **45**, 187-195 (2001).

9 Li, X. Z., Barre, N. & Poole, K. Influence of the MexA-MexB-oprM multidrug efflux system on expression of the MexC-MexD-oprJ and MexE-MexF-oprN multidrug efflux systems in Pseudomonas aeruginosa. *J Antimicrob Chemother* **46**, 885-893 (2000).

10 Guenard, S. *et al.* Multiple mutations lead to MexXY-OprM-dependent aminoglycoside resistance in clinical strains of Pseudomonas aeruginosa. *Antimicrob Agents Chemother* **58**, 221-228, doi:10.1128/AAC.01252-13 (2014).

11 Marvig, R. L., Sommer, L. M., Molin, S. & Johansen, H. K. Convergent evolution and adaptation of Pseudomonas aeruginosa within patients with cystic fibrosis. *Nat Genet* **47**, 57-64, doi:10.1038/ng.3148 (2015).

12 Greipel, L. *et al.* Molecular Epidemiology of Mutations in Antimicrobial Resistance Loci of Pseudomonas aeruginosa Isolates from Airways of Cystic Fibrosis Patients. *Antimicrob Agents Chemother* **60**, 6726-6734, doi:10.1128/AAC.00724-16 (2016).

13 Smith, E. E. *et al.* Genetic adaptation by Pseudomonas aeruginosa to the airways of cystic fibrosis patients. *Proc Natl Acad Sci U S A* **103**, 8487-8492, doi:10.1073/pnas.0602138103 (2006).

14 Campo Esquisabel, A. B., Rodriguez, M. C., Campo-Sosa, A. O., Rodriguez, C. & Martinez-Martinez, L. Mechanisms of resistance in clinical isolates of Pseudomonas aeruginosa less susceptible to cefepime than to ceftazidime. *Clin Microbiol Infect* **17**, 1817-1822, doi:10.1111/j.1469-0691.2011.03530.x (2011).

15 Wei, Q. *et al.* Phenotypic and genome-wide analysis of an antibiotic-resistant small colony variant (SCV) of Pseudomonas aeruginosa. *PLoS One* **6**, e29276, doi:10.1371/journal.pone.0029276 (2011).

**Supplementary Table and Legends**

**Supplementary Table S1**

| **Clone type** | **Mutation type** | **Mutated AA^1^** | **Affected domain^2^** | **Effect^3^** |
| --- | --- | --- | --- | --- |
| DK01 | Missense/Non-synonymous | R65H | CTD | Expected LOF |
| DK03 | Missense/Non-synonymous | V43A | DBD | Expected LOF |
| DK03 | Deletion/Frameshift |  | CTD | Expected LOF |
| DK04 | Missense/Non-synonymous | L163P | CTD | Expected LOF |
| DK06 | Deletion |  | DBD | Expected LOF |
| DK07 | Deletion/Frameshift |  | CTD | Expected LOF |
| DK07 | Insertion |  | CTD | Expected LOF |
| DK07 | Insertion/Frameshift |  | CTD | Expected LOF |
| DK08 | Deletion/Frameshift |  | CTD | Expected LOF |
| DK09 | Deletion/Frameshift |  | DBD | Expected LOF |
| DK09 | Insertion |  | CTD | Expected LOF |
| DK09 | Deletion/Frameshift |  | CTD | Expected LOF |
| DK14 | Deletion/Frameshift |  | CTD | Expected LOF |
| DK14 | Insertion/Frameshift |  | CTD | Expected LOF |
| DK15 | Deletion/Frameshift |  | CTD | Validated LOF^4^ |
| DK17 | Missense/Non-synonymous | Q10P | DBD | Expected LOF |
| DK19 | Missense/Non-synonymous | T177I | CTD | Validated LOF^4^ |
| DK19 | Deletion/Frameshift |  | CTD | Validated LOF^4^ |
| DK19 | Deletion/Frameshift |  | CTD | Expected LOF |
| DK19 | Deletion |  | CTD | Expected LOF |
| DK19 | Deletion/Frameshift |  | CTD | Expected LOF |
| DK19 | Deletion |  | CTD | Expected LOF |
| DK25 | Insertion/Frameshift |  | CTD | Expected LOF |
| DK26 | Deletion/Frameshift |  | CTD | Expected LOF |
| DK26 | Insertion |  | CTD | Expected LOF |
| DK26 | Deletion/Frameshift |  | CTD | Expected LOF |
| DK27 | Deletion/Frameshift |  | CTD | Expected LOF |
| DK31 | Deletion/Frameshift |  | CTD | Expected LOF |
| DK32 | Missense/Non-synonymous | R3G | DBD | Expected LOF |
| DK35 | Insertion/Frameshift |  | CTD | Expected LOF |
| DK36 | Missense/Non-synonymous | V43G | DBD | Expected LOF |
| DK36 | Missense/Non-synonymous | V48A | DRH | Expected LOF |
| DK36 | Missense/Non-synonymous | V105A | CTD | Expected LOF |
| DK36 | Deletion/Frameshift |  | CTD | Expected LOF |
| DK36 | Deletion/Frameshift |  | CTD | Expected LOF |
| DK36 | Deletion |  | CTD | Expected LOF |
| DK36 | Deletion/Frameshift |  | CTD | Expected LOF |
| DK41 | Missense/Non-synonymous | G46C | DRH | Expected LOF |
| DK41 | Deletion/Frameshift |  | CTD | Validated LOF^4^ |
| DK44 | Deletion/Frameshift |  | CTD | Expected LOF |

^1^ Mutated AA = Mutated Amino Acid

^2^ Affected domain in MexZ; DBD = DNA binding-domain; DRH = DNA-recognition helix; CTD = C-terminal domain.

^3^ Predicted or validated effect of mutation for MexZ function; Expected LOF = Expected loss of function; Validated LOF = Validated loss of function.

^4^ Validated loss of function mutations by qPCR; see Figure 2 and Supplementary Table 2.

**Supplementary Table S2**

| **Clonetype** | **Patient** | **Isolate number** | ***mexZ* phenotype^1^** | **Minimal inhibition concentration^2^** | | |
| --- | --- | --- | --- | --- | --- | --- |
|  |  |  |  | Tobramycin | Ciprofloxacin | Gentamicin |
| DK15 | Patient 7 | #1 | INDEL | 0.75 | 0.75 | 0.75 |
|  |  | #2 | INDEL | 1 | 2 | 1.5 |
|  |  | #3 | INDEL | 1 | 1.5 | 1.5 |
| DK19 | Patient 9 | #1 | Wild-type | 1.5 | 0.094 | 1.5 |
|  |  | #2 | SNP | 1 | 2 | 1.5 |
|  |  | #3 | INDEL | 1 | 0.25 | 2 |
|  |  | #4 | INDEL | 0.75 | 0.38 | 1 |
| DK41 | Patient 13 | #1 | INDEL | 3 | 2 | 4 |
|  |  | #2 | INDEL | 1 | 0.5 | 3 |

Table S2. MIC determination for clinical *P. aeruginosa* strains.

1. INDEL = Insertion/deletion mutation in *mexZ*; SNP = Single-nucleotide mutation in *mexZ*; wild-type = no mutations in *mexZ*.

2. Minimal inhibition concentration (MIC) determined with E-tests on Müller-Hinton agar.

**Supplementary Table S3**

| Strains | Relevant characteristics | Abbreviated form | Reference |
| --- | --- | --- | --- |
| *E. coli* DH_5_ɑ | Cloning host |  | ^4^ |
| *P. aeruginosa* PAO1 | Wild-type | PAO1 | ^5^ |
| *P. aeruginosa* PAO1 Δ*mexZ* |  | PAO1 Δ*mexZ* | This study |
| *P. aeruginosa* PAO1 Δ*mexY* |  | PAO1 Δ*mexY* | This study |
| *P. aeruginosa* PAO1 Tn7:*gfp* |  | PAO1 GFP | This study |
| *P. aeruginosa* PAO1 Tn7:*yfp* |  | PAO1 YFP | This study |
| *P. aeruginosa* PAO1 Δ*mexZ* Tn7:*cfp* |  | PAO1 Δ*mexZ* CFP | This study |
| Plasmids |  |  |  |
| RK600 | Cm^r^ *ori*-ColE1 RK2-mob^+^ RK2-tra^+^; helper plasmid in matings |  | ^6^ |
| pUX-BF13 | Ap^r^ mob^+^ *ori*-R6K; helper plasmid; providing the tn7 transposition functions in trans |  | ^7^ |
| pBK-mini-Tn7-*gfp2* | Gm^r^ Ap^r^ mob^+^; delivery plasmid for mini-Tn7-Gm^r^-P_A1/04/03_-egfp |  | ^8^ |
| pBK-mini-Tn7-*yfp* | Gm^r^ Ap^r^ mob^+^; delivery plasmid for mini-Tn7-Gm^r^-P_A1/04/03_-eyfp |  | ^8^ |
| pBK-mini-Tn7-*cfp* | Gm^r^ Ap^r^ mob^+^; delivery plasmid for mini-Tn7-Gm^r^-P_A1/04/03_-ecfp |  | ^8^ |
| pEX19Gm | Gm^r^ *oriT* *sacB* *lacZα* MCS from pUC19; cloning plasmid |  | ^2^ |
| pXZL34 | *oprM* expression vector |  | ^9^ |
| pJFM20 | pEX19Gm with PAO1 *mexZ* flanking regions inserted into the MCS |  | This study |
| pJFM21 | pEX19Gm with PAO1 *mexY* flanking regions inserted into the MCS |  | This study |

Table S3. Strains and plasmids.

**Supplementary Table S4**

| Primer | Sequence (5’-3’) | Source |
| --- | --- | --- |
| *mexZ*_PAO1_UP_FW | AGCTCGGAAUTCGCGATGCGGATTGCGG | This study |
| *mexZ*_PAO1_UP_Rv | ACTGCGUGAACGTCCTCACAAGGGAAA | This study |
| *mexZ*_PAO1_DW_Fw | ACGCAGUTCTCCCTACCTGTTGCTGGCG | This study |
| *mexZ*_PAO1_DW_Rv | AAGGAAAUCTTGGTGGCGAGGAAGGCATT | This study |
| *mexY*_PAO1_UP_Fw | AAAGTTCUCCCTGGGCAGTGCAAACAAG | This study |
| *mexY*_PAO1_UP_Rv | ACGCTACGAUAGCGAAACTCTCGCTCCCC | This study |
| *mexY*_PAO1_DW_Fw | ATCGTAGCGUTCTCCGTCACTGGCCCGCCG | This study |
| *mexY*_PAO1_DW_Rv | ATTATTCUCCCAGCCGGCCGGCGAAGT | This study |
| pEX19Gm_*mexZ*_PAO1_Fw | ATTTCCTUCGCGTAATCATGGTCATAGCTGTT | This study |
| pEX19Gm_*mexZ*_PAO1_Rv | ATTCCGAGCUCGAATTCACTGGCCGTCGTT | This study |
| pEX19Gm_*mexY*_PAO1_Fw | AGAATAAUCATGGTCATAGCTGTTTCCT | This study |
| pEX19Gm_*mexY*_PAO1_Rv | AGAACTTUGGCCGTCGTTTTACAACGT | This study |
| *mexZ*_qPCR_Fw | CGGTCTACGGCCACTACAAG | This study |
| *mexZ*_qPCR_Rv | CAACAGCGGCTCGTTCTC | This study |
| *mexY*_qPCR_Fw | CCGCTACGACGCCATGG | This study |
| *mexY*_qPCR_Rv | GTGAGGCGGGCGTTGTG | This study |
| *oprM*_qPCR_Fw | CTTCACCGAGCAGTTGCAG | This study |
| *oprM*_qPCR_Rv | GAGGGCCTTGTACAGGTTGA | This study |
| *rplS*_qPCR_Fw | GCAACTATCAACCAGCTGGTG | ^10^ |
| *rplS*_qPCR_Rv | GCTGTGCTCTTGCAGGTTGTG | ^10^ |
| 16S_f | TGTCGTCAGCTCGTGTCGTGA |  |
| 16S_r | ATCCCCACCTTCCTCCGGT |  |

Table S4. Primers used in this study.

**Supplementary Figures**

**Figure S1.** *Distribution of mutations in MexZ protein sequence*. Lollipop plot showing the location (needle) and effect (dot/segment color) of genetic mutations identified in Marvig *et al.*^11^ and discussed in this work (main plot). Segments plotted over the domain structures of MexZ protein represents mutations identified in previous work (Gr: Greipel *et al.*^12^; Sm: Smith *et al.*^13^; Gu: Guénard *et al.*^10^; O: Esquisabel *et al.^14^* and Wei *et al.*^15^).

**Figure S2.** *Patient treatment regime.* For each patient their treatment regime and acquisition of mutations in *mexZ* are given over time. The top panel shows which antibiotic the patient was treated with and the length of the treatment; AZT = Aztreonam, CEF = Ceftazidim, CIP = Ciprofloxacin, MER = Meropenem, TAZ = Tazocin (piperacillin/tazobactam), TOB = Tobramycin. The lower panel shows the *mexZ* allele from each isolated patient sample plotted over time; non-sequenced *P. aeruginosa* (◊), wild-type allele (•), SNP in *mexZ* (□), and INDEL in *mexZ* (Δ). If more than one unique INDEL and/or SNP exist within the *P. aeruginosa* population the triangle (Δ) or square (□), respectively, is modified from each other to distinguish the unique clones within the lineage. Each individual graph presents a *P. aeruginosa* clone-type colonizing one specific CF patient. Graphs are only shown for patients with more than four *P. aeruginosa* isolates with a *mexZ* mutation.

**Figure S3.**  *Fixation of the mexZ mutation in P. aeruginosa airway population of CF patients.* The *mexZ* allele from each isolated patient sample is plotted over time along with the accompanying MIC to the MexXY-OprM substrate tobramycin tested by E-test; wild-type allele (•), SNP in *mexZ* (□), and INDEL in *mexZ* (Δ). If more than one unique INDEL and/or SNP exist within the *P. aeruginosa* population the triangle (Δ) or square (□), respectively, is modified from each other to distinguish the unique clones within the lineage. Each individual graph presents a *P. aeruginosa* clone-type colonizing one specific CF patient. Graphs are only shown for patients with more than four *P. aeruginosa* isolates with a *mexZ* mutation. The EUCAST break-off point for tobramycin resistance (4 µg/mL) is noted by a horizontal line at the break-off point.

**Figure S4**. *Phenotypic variation in carbon metabolism of PAO1 and PAO1 ΔmexZ*. PM kinetics from PM1 and PM2a plates for wells with different carbon sources in them. The PM kinetic results show consensus data comparing the wild-type (green) with Δ*mexZ* (red). When the two strains reveal an equivalent activity or equivalent growth response in a well, the kinetic graphs overlap and are yellow. Red kinetic graphs indicate a stronger response by Δ*mexZ*, and green kinetic graphs a stronger response by the wild-type. (A) PAO1 (green) vs PAO1 Δ*mexZ* (red) for growth in the PM1 plate; (B) PAO1 (green) vs PAO1 Δ*mexZ* (red) for growth in the PM2a plate.

Figure S1.


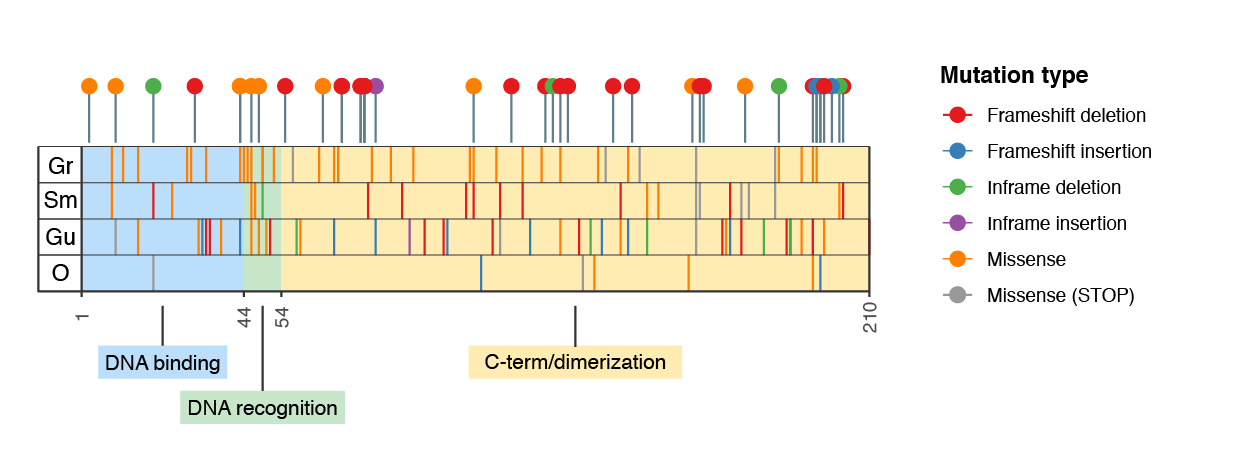


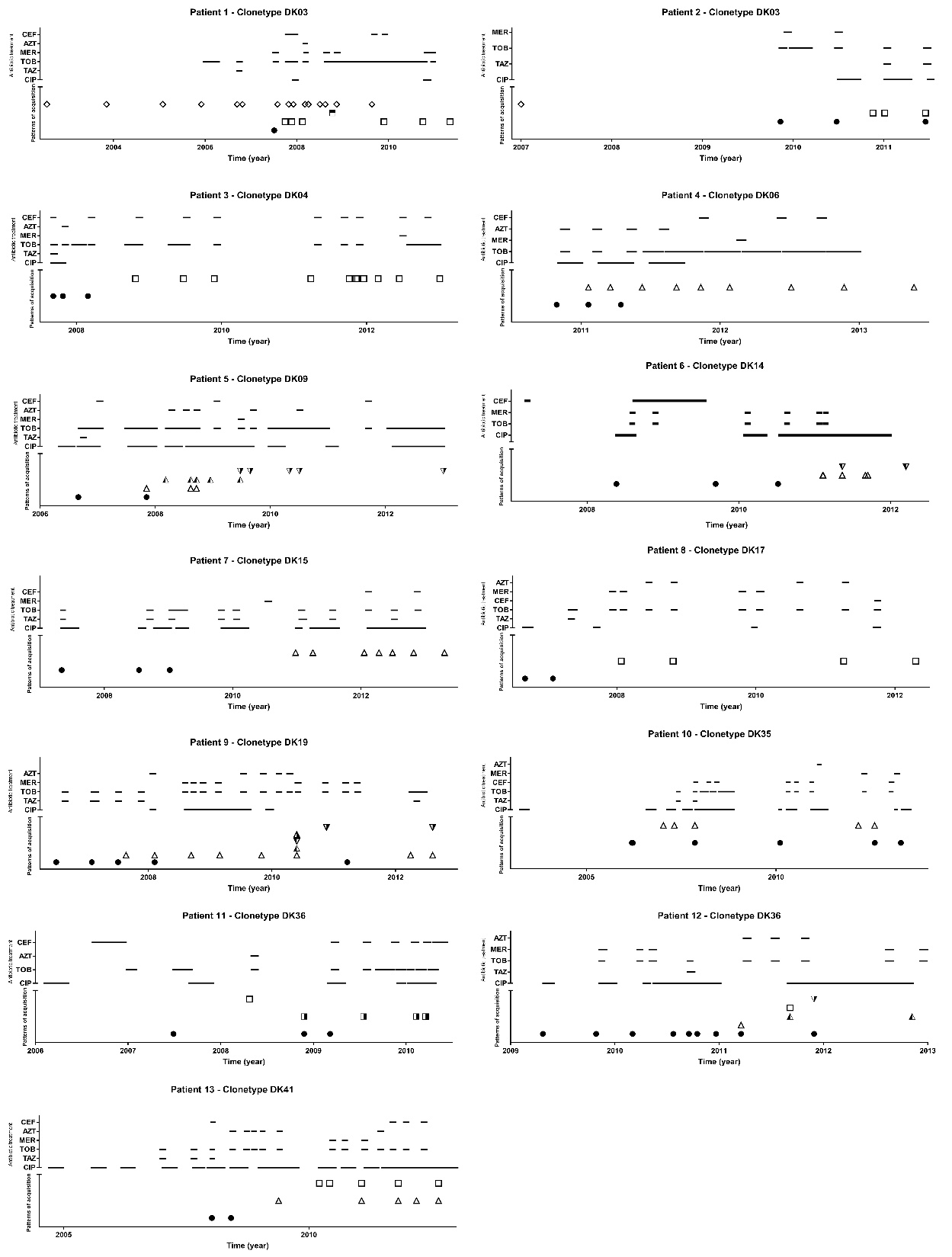
Figure S2.

Figure S3.


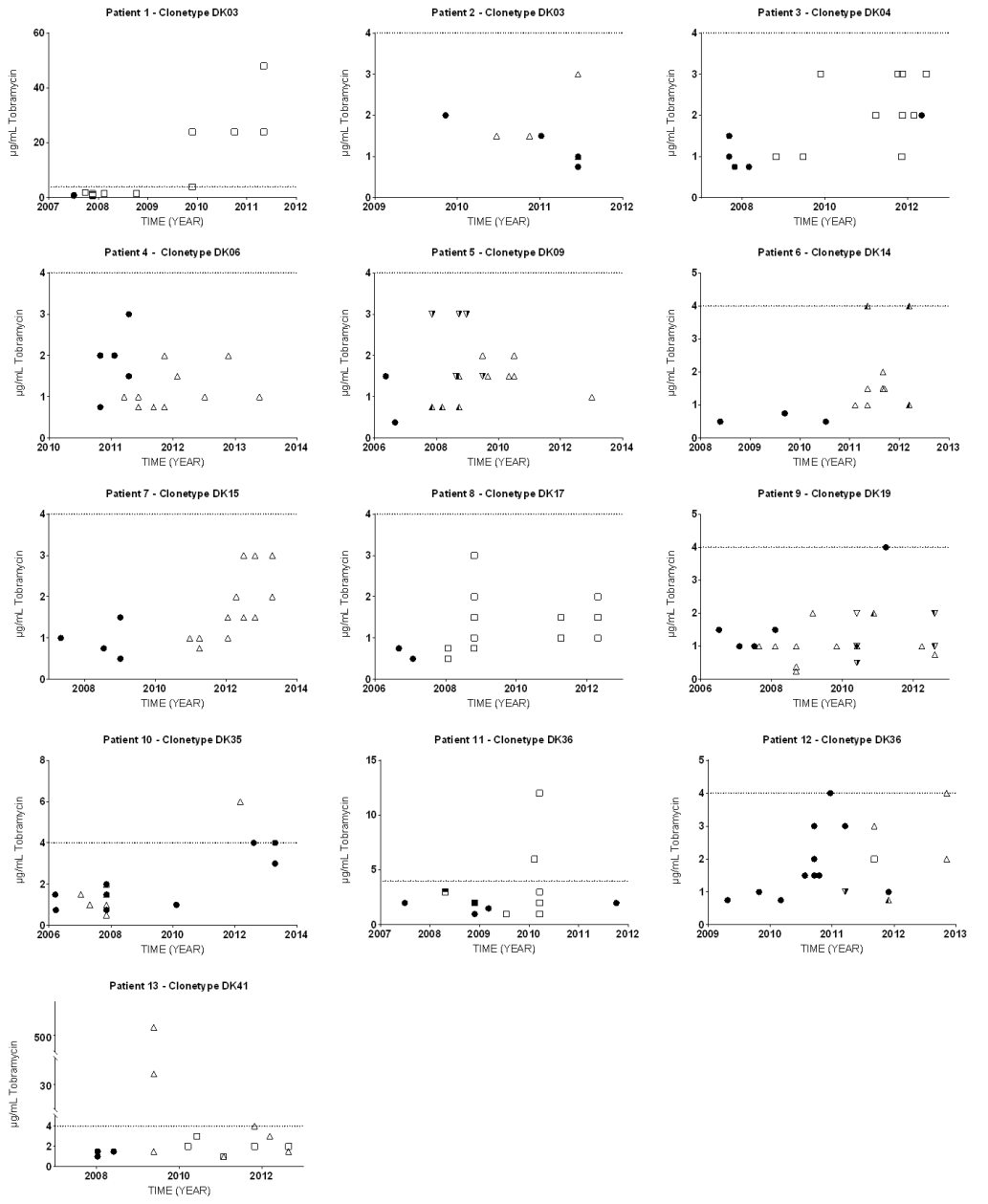


Figure S4.

1. PAO1 vs PAO1 Δ*mexZ*. Biolog plate PM1.


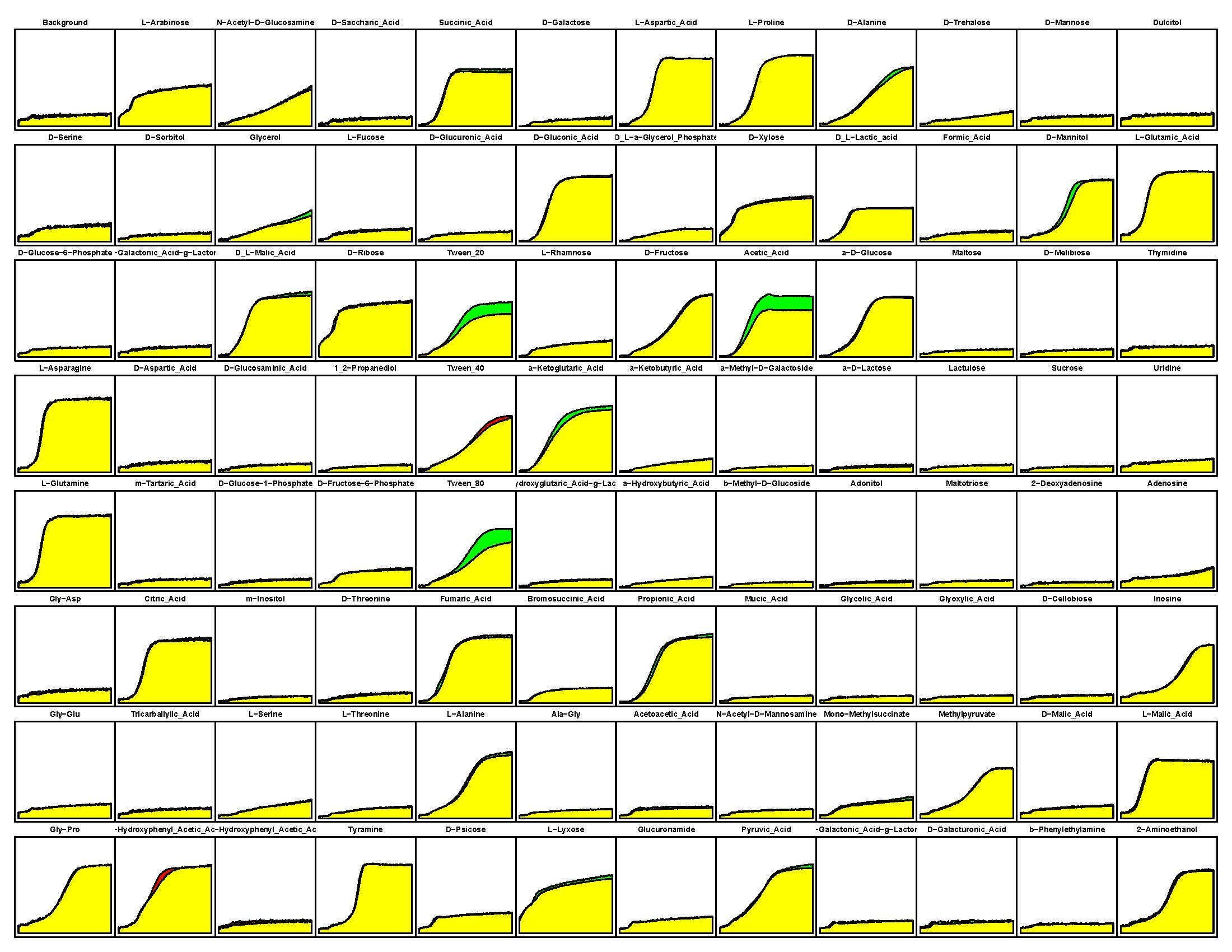


1. PAO1 vs PAO1 Δ*mexZ*. Biolog plate PM2.


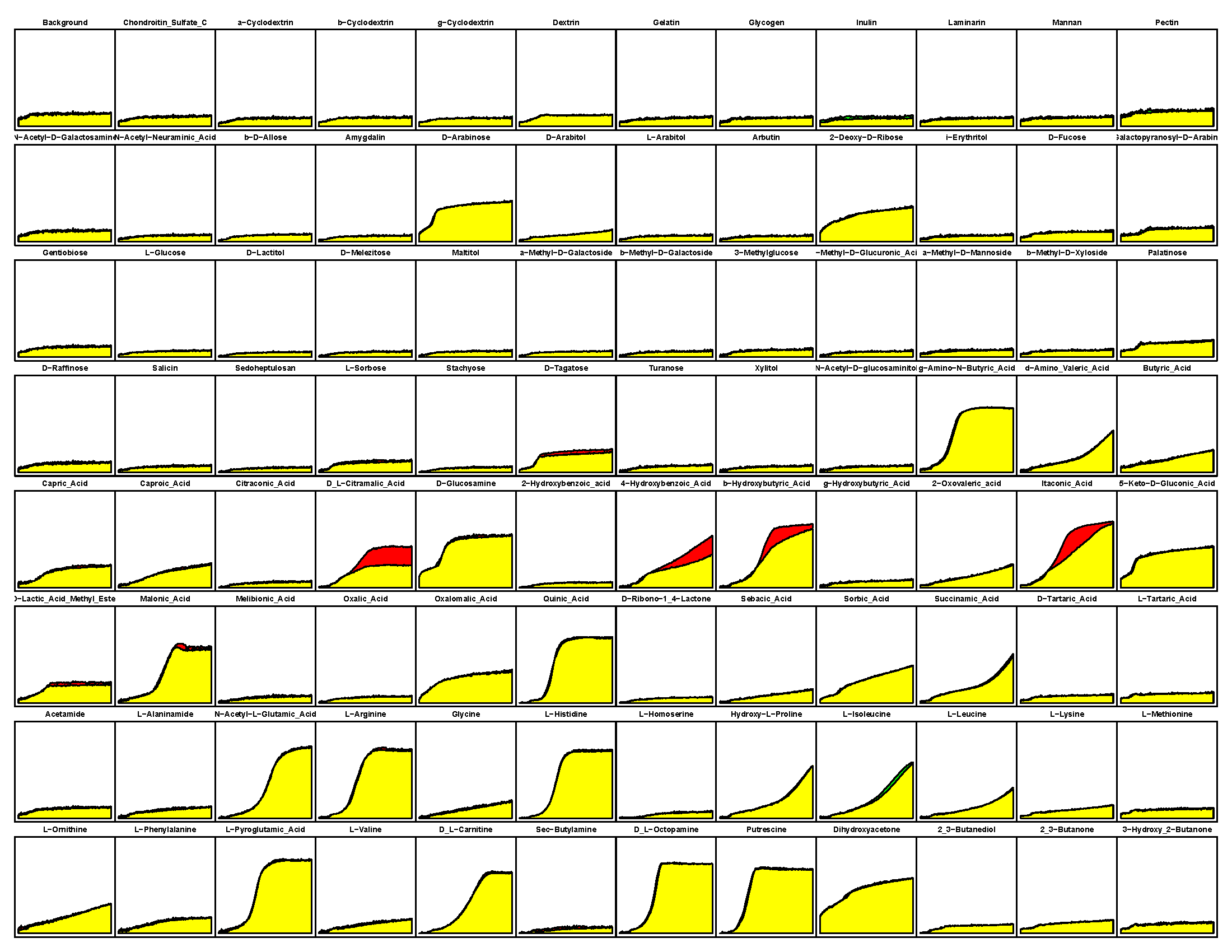

Supplement: Supplementary file 1 — Supplementary material and methods [file 41598_2018_30972_MOESM1_ESM.docx]
